# Retinal biomarkers and pharmacological targets for Hermansky-Pudlak syndrome 7

**DOI:** 10.1038/s41598-020-60931-5

**Published:** 2020-03-04

**Authors:** Giovanni Luca Romano, Chiara Bianca Maria Platania, Gian Marco Leggio, Sebastiano Alfio Torrisi, Salvatore Giunta, Salvatore Salomone, Michele Purrello, Marco Ragusa, Cristina Barbagallo, Frank J. Giblin, Rosa Mastrogiacomo, Francesca Managò, Maurizio Cammalleri, Francesco Papaleo, Filippo Drago, Claudio Bucolo

**Affiliations:** 1 Bascom Palmer Eye Institute, University of Miami Miller School of Medicine, Miami, USA; 2 Department of Biomedical and Biotechnological Sciences, School of Medicine, University of Catania, Catania, Italy; 3 Center for Research in Ocular Pharmacology-CERFO, University of Catania, Catania, Italy; 4 Oasi Research Institute - IRCCS, Troina, Italy; 5 Eye Research Institute, Oakland University, Rochester, Michigan USA; 6 Genetics of Cognition Laboratory, Department of Neuroscience and Brain Technologies, Istituto Italiano di Tecnologia, Genova, Italy; 7 Department of Biology, University of Pisa, Pisa, Italy

**Keywords:** Predictive markers, Hereditary eye disease

## Abstract

Deletion of dystrobrevin binding protein 1 has been linked to Hermansky-Pudlak syndrome type 7 (HPS-7), a rare disease characterized by oculocutaneous albinism and retinal dysfunction. We studied dysbindin-1 null mutant mice (Dys^−/−^) to shed light on retinal neurodevelopment defects in HPS-7. We analyzed the expression of a focused set of miRNAs in retina of wild type (WT), Dys^+/−^ and Dys^−/−^ mice. We also investigated the retinal function of these mice through electroretinography (ERG). We found that miR-101-3p, miR-137, miR-186-5p, miR-326, miR-382-5p and miR-876-5p were up-regulated in Dys^−/−^mice retina. Dys^−/−^ mice showed significant increased b-wave in ERG, compared to WT mice. Bioinformatic analysis highlighted that dysregulated miRNAs target synaptic plasticity and dopaminergic signaling pathways, affecting retinal functions of Dys^−/−^ mice. Overall, the data indicate potential mechanisms in retinal neurodevelopment of Dys^−/−^ mice, which may have translational significance in HSP-7 patients, both in terms of diagnostic/prognostic biomarkers and novel pharmacological targets.

## Introduction

Dystrobrevin binding protein 1 gene (*DTNBP1*) encodes dysbindin-1, a ubiquitous protein that regulates membrane localization of synaptic proteins, through the regulation of synaptic vesicles and receptors recycling^1,2^. Dysbindin-1 is widely expressed in the brain, both in neurons and glial cells^1,3–5^. Dysbindin-1 is also expressed in the eye^6^ and mutations leading to DTNBP1 deletion have been associated with the subtype 7 of Hermansky-Pudlak syndrome (HPS-7)^7,8^.

Hermansky-Pudlak syndromes (HPS) are heterogeneous genetic disorders characterized by pulmonary fibrosis, abnormalities in platelet aggregation and oculocutaneous albinism^7,8^. Pulmonary fibrosis is the most serious complication of HPS, which cannot be effectively treated with steroids or pirfenidone, when insufficient residual lung function occurs^9^.

The zebrafish *fade out* (*fad*) locus mutant has been reported as lower vertebrate model of HSP with retinal morphology and function characterization^10^. Recently, *DTNBP1* knock-out mice (Dys^−/−^) showed ocular albinism related to a drop out of melanosomes in retinal pigmented epithelium and choroid, compared to wild type mice (WT)^11^. Additionally, retinal melanosomes in Dys^−/−^ mice were found to have irregular shape and small dimensions^7^.

Retinal function has not yet been evaluated in Dys^−/−^ mice. Visual dysfunction, i.e. decreased ERG response was found in patients with HPS syndromes^12^ and ocular albinism^13^. Interestingly, visual dysfunctions have also been found in schizophrenic patients and individuals bearing dysbindin mutations, associated with increased risk of schizophrenia development^6,14^. Furthermore, several mutations at *DTNBP1* have been associated with human intelligence^15–18^, and cognitive responses to antipsychotics in animal models and patients with schizophrenia^19,20^. Dysregulation of dysbindin-1 expression and function, related to gene mutation, have detrimental effects on neurodevelopment^1^.

On the premises that the eye is the embryonic extension of the brain^21^ and ocular albinism has been associated to neurodevelopmental disorders^22,23^, we focused our research on the effects of dysbindin-1 deletion in the retina, by using Dys^−/−^ mice as a novel animal model of HPS-7. Because the role of dysbindin in neurodevelopment has been widely investigated, we also explored the effect of dysbindin mutation on miRNAs expression; this kind of approach has been previously used on several animal paradigms such as fragile X mental retardation protein knockout mice (FMRP^−/−^)^24^, apolipoprotein E-knockout mice (APOE^−/−^)^25^, PSEN1/PSEN2 double knockout^26^, PTEN knockout mice^27^.

Expression analysis of 13 miRNAs, a selected set obtained from preliminary *in-silico* analysis, was carried out in retina and serum of Dys^+/−^, Dys^−/−^ and WT mice. On the basis of ERG abnormalities previously found in children and young adults with ocular albinism^13^ and HPS syndromes^12^, ERG analysis on Dys^−/−^ mice was also carried out. We identified 6 out of 13 miRNAs (miR-101-3p, miR-137, miR-186-5p, miR-326, miR-382-5p and miR-876-5p) up-regulated in Dys^−/−^ mice retina. Accordingly, abnormalities in ERG b-wave amplitude were detected in Dys^−/−^ mice. This study was aimed at identification of novel miRNAs as prognostic/diagnostic tools for HPS-7, as well as, new potential pharmacological targets for HPS-7 complications, such as pulmonary fibrosis.

## Results

### Prediction of miRNA dysregulated in Dys^−/−^ mice

High-throughput expression analysis of miRNAs is expensive and may require large number of samples^28^; for this reason, we first carried out an integrated bioinformatic approach to select miRNAs potentially dysregulated in Dys^−/−^ mice (Tables 1 and 2). With this bioinformatic approach we mined 40 miRNAs. We also predicted the pathways^29^ that can be dysregulated by these retrieved miRNAs. Subsequently, miRNAs were rescored based on their regulatory potential on pathways linked to albinism or neurodevelopment (see methods section). After rescoring, the expression of the top scored 13 miRNAs (miR-377-3p, miR-876-5p, miR-224-5p, miR-326, miR-330-5p, miR-155-5p, miR-590-3p, miR-101-3p, miR-137, miR-186-5p, miR-382-5p, miR-146a-5p, and miR-27a-3p) was evaluated in retina and serum of Dys^−/−^, Dys^+/−^ and WT mice.

**Table 1:** Prediction of miRNA-binding sites modification upon dysbindin gene mutations.

miRNA	SNP (DTNBP1)	mirSVR	Effect
miR-1193	rs742106		Create
miR-1246	rs742106	−0.500	Enhance
miR-1293	rs742106	−1.014	Break
miR-3167	rs742106	−0.833	Enhance
miR-377-3p	rs1047631	−1.072	Decrease
miR-4275	rs1047631		Create
miR-432-3p	rs742106		Create
miR-4483	rs742106		Break
miR-4495	rs1047631		Break
miR-4511	rs2056943		Create
miR-4694-3p	rs742106		Enhance
miR-4760-3p	rs2056943		Break
miR-4782-5p	rs742106		Create
miR-5706	rs742106		Create
miR-876-5p	rs742106	−0.830	Decrease

**Table 2:** Prediction of miRNAs targeting genes associated with albinism.

Genes	Top scored miRNAs
Melanine Biosynthesis	
*TYR*	miR-326, miR-330-5p, miR-328, miR-506, miR-124, miR-378
*TYRP1*	miR-155, miR-590-3p, miR-128, miR-154, miR-365
*OCA2*	miR-495, miR-101, miR-590-3p, miR-212
*SLC45A2*	miR-154
**Melanocyte development**	
*DTNBP1*	miR-224
*PAX3*	miR-1, miR-206, miR-613
*MITF*	miR-137, miR-186, miR-152
*SRY*	miR-209, miR-219-5p, miR-487, miR-155
*SOX10*	miR-590-3p, miR-221, miR-222
*EDNRB*	miR-590-5p (3p), miR-382, miR-146a
*EDN3*	miR-496, miR-186, miR-27a

### Expression pattern of miRNAs in serum and retina

**Figure 1:**
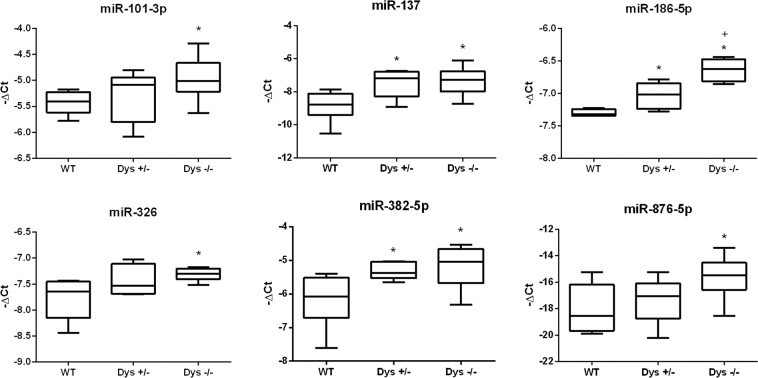
miRNAs dysregulated in the retina of WT, Dys^+/−^ and Dys^−/−^ mice. −ΔCt distribution box-plot. *p < 0.05 Dys^+/−^ or Dys^−/−^ *vs*. WT mice; +p < 0.05 Dys^−/−^ *vs*. Dys^+/−^ mice (N = 6 mice per group, each run in triplicate).

We evaluated the expression of miR-377-3p, miR-876-5p, miR-224-5p, miR-326, miR-330-5p, miR-155-5p, miR-590-3p, miR-101-3p, miR-137, miR-186-5p, miR-382-5p, miR-146a-5p, and miR-27a-3p in serum and retina of WT, Dys^+/−^ and Dys^−/−^ mice. Expression analysis of these miRNAs in serum did not show any significative difference between experimental groups (data not shown). On the contrary, we found 6 out of 13 miRNA up-regulated in retina of Dys^−/−^ compared to WT mice: miR-101-3p, miR-137, miR-186-5p, miR-326, miR-382-5p and miR-876-5p (Fig. 1). Moreover, dysbindin deletion in mutant mice (Dys^+/−^ and Dys^−/−^) led to gene dose effect on miRNAs dysregulation, which attained statistical significance for miR-186-5p. Moreover, a loss of one copy of the dysbindin gene significantly led to increased expression of miR-137 and miR-382-5p in Dys^+/−^, compared to WT mice.

### Post-hoc bioinformatic analysis

In order to understand the biological impact of dysregulated miRNAs in the Dys^−/−^ retina, we analyzed potential miRNA-pathway interactions by accessing to the DIANA miRPath v. 3 tool, using as input miR-101-3p, miR-137, miR-186-5p, miR-326, miR-382-5p, and miR-876-5p and iteratively applying the Tarbase, microT-CDS and Targetscan prediction algorithms^30^. On the basis of the experimentally validated miRNA:mRNA interactions, the application of the Tarbase algorithm to the DIANA miRPath v. 3 tool gave a list of pathways regulated only by miR-101-3p, miR-186-5p, and miR-382-5p (Table 1S, supplemental information). These results are consistent with the main function of dysbindin, in fact, miR-101-3p, miR-186-5p and miR-382-5p were predicted to control “endocytosis” and “protein processing in the endoplasmatic reticulum”. Furthermore, miR-101-3p and miR-186-5p can control the expression of genes belonging to the “melanogenesis” pathway (p-value 0.033, Fig. 1S, supplemental information). Interestingly, Tarbase prediction (Table 1S, supplemental information) showed that target genes of miR-101-3p and miR-186-5p control the expression of genes belonging to the “long term potentiation” (LTP) and “long term depression” (LTD) pathways. This result is consistent with evidences about the role of dysbindin in synaptic plasticity^31^. In order to predict which pathways can be targeted by all 6 microRNAs up-regulated in the Dys^−/−^ retina, microT-CDS and Targetscan algorithms were further applied; data are shown in Tables 3 and 4, respectively.

**Table 3:** Pathways dysregulated in the Dys^−/−^ retina – microT-CDS prediction.

KEGG pathway	p-value	#genes	#miRNAs
Prion diseases	1.44E-17	3	3
MAPK signaling pathway	2.68E-07	58	6
Axon guidance	0.000285	32	6
**Endocytosis**	0.000792	38	6
**Long-term potentiation**	0.001331	20	5
Rap1 signaling pathway	0.001331	41	6
Thyroid hormone signaling pathway	0.001403	23	6
Oxytocin signaling pathway	0.001742	32	6
mRNA surveillance pathway	0.002901	22	6
Calcium signaling pathway	0.003031	35	6
mTOR signaling pathway	0.00307	18	6
Fatty acid elongation	0.015193	4	3
**Glutamatergic synapse**	0.015193	21	5
HTLV-I infection	0.015193	47	6
Adherens junction	0.017904	15	6
cGMP-PKG signaling pathway	0.017904	33	6
FoxO signaling pathway	0.017904	30	6
Wnt signaling pathway	0.020194	28	6
cAMP signaling pathway	0.022403	37	6
T cell receptor signaling pathway	0.022579	22	6
Ubiquitin mediated proteolysis	0.024099	29	6
VEGF signaling pathway	0.024486	15	5
Insulin signaling pathway	0.024486	28	6
Bacterial invasion of epithelial cells	0.024486	17	6
Transcriptional misregulation in cancer	0.026035	31	6
Neurotrophin signaling pathway	0.026125	24	6
Vasopressin-regulated water reabsorption	0.026702	8	6
Amyotrophic lateral sclerosis (ALS)	0.026758	13	6
**Protein processing in endoplasmic reticulum**	0.034352	29	6
**Amphetamine addiction**	0.040134	15	4
AMPK signaling pathway	0.041784	24	6
**TGF-beta signaling pathway**	0.041784	20	5
Circadian entrainment	0.045922	18	6

**Table 4:** Pathways dysregulated in the Dys^−/−^ retina – Targetscan prediction.

KEGG pathway	p-value	#genes	miRNAs
Adherens junction	3.03E-05	10	miR-101-3p/miR-326
**Morphine addiction**	0.010632	7	miR-101-3p/miR-326
Notch signaling pathway	0.014221	9	miR-326
Endocytosis	0.014221	20	miR-101-3p/miR-326

**Figure 2:**
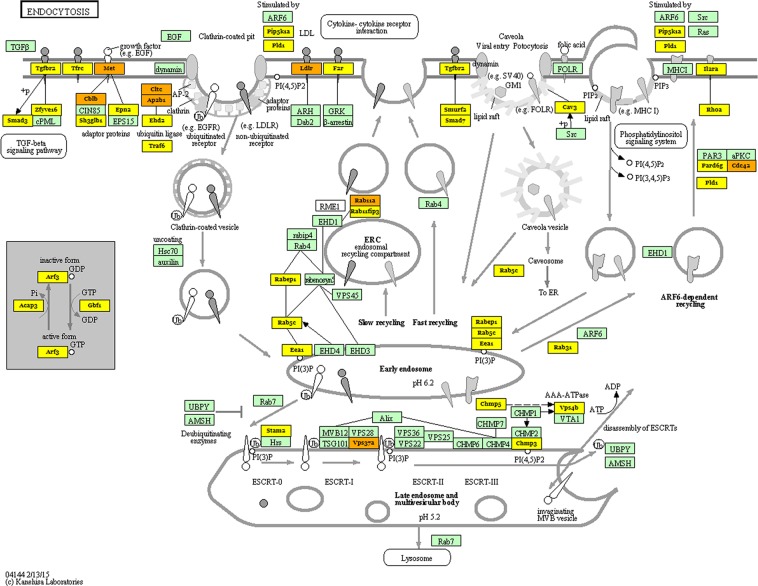
Genes belonging to the “endocytosis pathway” are targets of miRNAs up-regulated in the Dys^−/−^ retina. Yellow genes are targets of one miRNA, while orange genes are targets of more than one miRNA. This picture is the output of Diana miRPath software and recalls the KEGG pathway deposited in the database (KEGG permission n°190309)^80^: https://www.genome.jp/kegg/pathway.html.

All three different algorithms predicted that miR-101-3p, miR-137, miR-186-5p, miR-326, miR-382-5p and miR-876-5p can modulate cellular endocytosis (Fig. 2), consistently with dysbindin main functions^32–34^.

**Figure 3:**
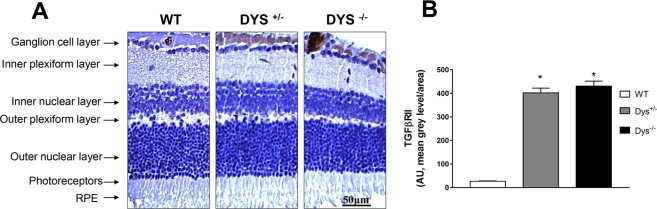
TGFβRII staining in the retinal ganglion cell layer. Representative IHC images (**A**) and densitometric analysis (**B**) for TGFβRII staining in the retinal ganglion cell layer of WT (white bars), Dys^+/−^ (gray bars) and Dys^−/−^ (black bars). Each column represents the average ± SD (N = 12). *p < 0.05 *vs*. WT.

Both Tarbase and microT-CDS predictions evidenced that TGF-β signaling pathway can be dysregulated by miRNAs up-regulated in the Dys^−/−^ retina. The microT-CDS algorithm predicted that miR-101-3p, miR-137, miR-186-5p, miR-326 and miR-382 can control the TGF-β signaling pathway, which is known to be involved in collagen 1 synthesis and induction of fibrosis^35^. This result may be relevant for pulmonary fibrosis, often occurring in HPS-7. Specifically, miR-101-3p (*TGFBRI*, *ACVR2*) and miR-186-5p experimentally target genes of the TGFβ signaling pathway (*ACVR1*, *SMAD4*, *SMAD5*, *ACVR2A*). Therefore, we tested the hypothesis that also the retina of Dys^−/−^ mice could be affected by a dysfunctional TGFβ signaling. To this aim, we stained the retina for TGFβ1, TGFβRI and TGFβRII. We found a significant (p < 0.05) increased staining for TGFβRII, but not for TGFβ1 and TGFβRI, in the retinal ganglion cell layer of Dys^+/−^ and Dys^−/−^ mice, compared to WT mice (Fig. 3).

Furthermore, targetscan and microT-CDS predictions suggested the interaction of dysregulated miRNAs in Dys^−/−^ mice with genes belonging to the pathways named “amphetamine addiction”, “morphine addiction” and “glutamatergic synapse”. These genes are listed in Table 5, and these results suggest that retinal dysregulation of these miRNAs may affect dopaminergic, GABAergic signaling and glutamatergic signaling, which were found to be involved in retinal function and retinal light adaptation^36,37^.

**Table 5:** Genes that are target of miRNAs dysregulated in retina of Dys^−/−^ mice (microT-CDS algorithm).

**Amphetamine addiction**
*SLC6A3, GRIN3A, CAMK2A, STX1A, PP1CA*, and *PDYN*
**Morphine addiction**
*OPRM1, GABRG3, GNB3, KGNJ5*, and *PDE7B*
**Glutamatergic synapse**
*PPP3CC, GRIA2, PPP3R2, SLC1A1, PPP3CB, ADCY1, SHANK2, HOMER2, GNB4, ADCY3, GRM5, PPP3R1, DLGAP1, PPP3CA, PLCB1, GRIK3, GNB3, SLC17A8, HOMER1*, and *SLC17A6*

### Scotopic ERG analysis

**Figure 4:**
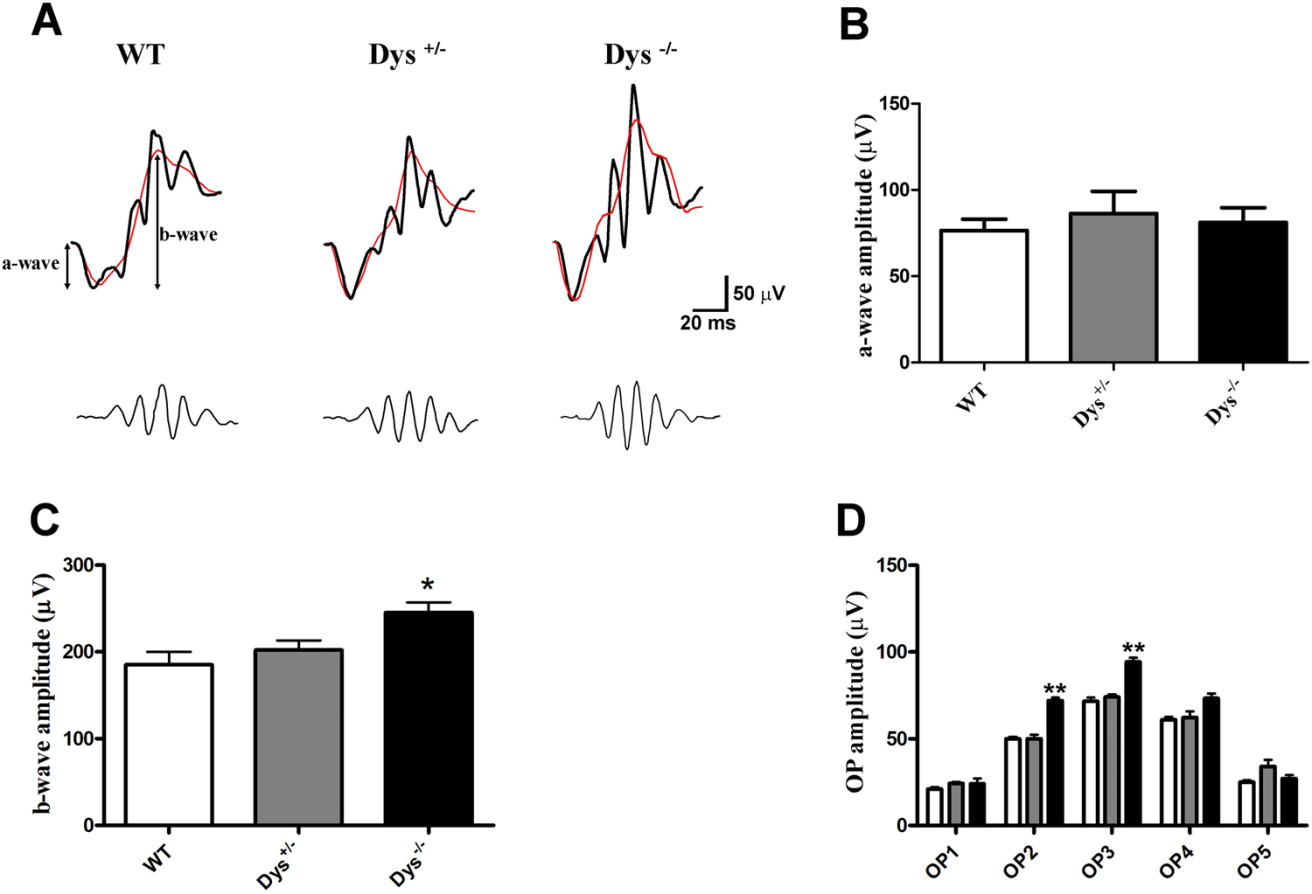
ERG records in wild type (WT), Dys^+/−^ and Dys ^−/−^ mice. Representative ERG waveforms (top) w/o OPs (black and red lines, respectively) and representative OP waveforms (bottom) (**A**), a-wave amplitudes (**B**), b-wave amplitudes (**C**), and OPs amplitude (**D**) in WT (white bars), Dys^+/−^ (gray bars) and Dys^−/−^ (black bars) mice recorded at light intensity of 1 log cd-s/m^2^. *p < 0.05 and **p < 0.01 *vs*. WT. Each column represents the average ± SD (N = 12).

ERG was recorded in mice belonging to the three experimental groups: WT, Dys^+/−^ and Dys^−/−^. The representative recordings in Fig. 4 (panel A) show scotopic ERG waveforms (a-wave, the b-wave and oscillatory potentials (OPs) on the rising part of the b-wave) recorded at light intensities of 1 log cd-s/m^2^. It can be noticed that the ERG amplitude was altered in Dys^−/−^ dark-adapted mice. Comparing the average amplitude of the a-wave (Fig. 4B) and b-waves (Fig. 4C) in controls WT, Dys^+/−^ and Dys^−/−^ mice, we found a statistically significant difference between b-wave amplitude records in WT *vs*. Dys^−/−^ mice (p < 0.05). Oscillatory potentials (OPs) represent high-frequency oscillations on the leading edge of the ERG b-wave. It should be noted that OP2 and OP3 in the Dys^−/−^ mice showed amplitudes larger than control animals, which may indicate alterations in the activity of outer and inner retina, respectively (Fig. 4D). Furthermore, the lack of dysbindin was associated with an increase in b-wave amplitude in mice, consistently with a previous report of increased b-wave amplitude in dark-adapted albinos^13^. Moreover, a-wave and b-wave abnormalities have been previously found in schizophrenic patients^38^ or individuals bearing high genetic risk of schizophrenia^39^.

## Discussion

Dysbindin-1 is widely expressed in the brain and plays a role in protein trafficking and synaptic transmission. Dysbindin alterations have been linked to altered neuronal plasticity attributed to its influence on neurodevelopment during the embryonal stage.

Dysbindin mutations have been associated to a rare disease, named Hermansky-Pudlak syndrome 7 (HPS-7), characterized by pulmonary fibrosis, abnormalities in platelet aggregation and oculocutaneous albinism, which alters retinal functions^13^.

We analyzed miRNA expression profiles and function in the retina of Dys^−/−^ mice, to shed light on pathological mechanisms and retinal defects upon deletion of dysbindin, which may have translational value for the Hermansky-Pudlak syndrome 7 (HPS-7). We first predicted with bioinformatic tools a small set of miRNAs, putatively involved in oculocutaneous albinism, and then the expression profile of these miRNAs was analyzed in the retina and serum of Dys^−/−^ mice. No significative differences were found in serum of Dys^−/−^ mice, compared to either Dys^+/−^ or WT mice (data not shown). On the contrary, 6 out of 13 miRNAs were found to be significantly up-regulated in Dys^−/−^ mouse retina, compared to WT or Dys^+/−^ groups. These data suggest that *DTNBP1* depletion can perturb a yet unknown molecular signaling pathway, which increases expression of a specific set of miRNAs potentially related to albinism and/or retinal neurodevelopment. It was previously found that *FMRN* gene deletion in mice led to the up-regulation of a series of miRNAs, likely influencing the degradation of pre-miRNAs instead of promoting their expression^24^. Moreover, BDNF was found to influence miRNA maturation and stability by modulation of DICER and lin28b expression^40^. Furthermore, gene deletion led to miRNAs dysregulation in several experimental paradigms^25–27^. Dysbindin is a component of BLOC-1, which mediates the sorting of lysosome-related organelles (LROs) and the lack of dysbindin leads to an altered sorting of lysosomes and melanosome maturation^7^. Additionally, Argonaute 2 (Ago2)-miRNA lysosomal sorting was found to be modulated by BLOC3 in *Drosofila*^41^. Thus, we hypothesize that the lack of dysbindin could also influence the sorting of miRNAs, leading to their up-regulation in retinal tissues.

Diana miRPath post-hoc analysis revealed that miRNAs that are up-regulated in the Dys^−/−^ mice retina are linked to “endocytosis” and “protein processing in the endoplasmatic reticulum”, which are pathways consistent with the functions of dysbindin mentioned above. Additionally, the bioinformatic analysis with Diana miRPath revealed that up-regulated miRNAs in Dys^−/−^ mice can dysregulate the TGFβ signaling pathway. This bioinformatic result might partially explain a feature of HPS-7, i.e. pulmonary fibrosis. In fact, the TGF-β signaling pathway, when over activated, leads to pro-fibrotic events and its involvement in pulmonary fibrosis has been widely reported^42–44^. Interestingly, the role of regulation of fibrosis by miR-101-3p and miR-326 was also reported in a recent review^35^. Moreover, the induced expression of miR-101-3p decreased signs of pulmonary fibrosis in an animal model^45^; while, miR-326 was found to be down-regulated in lung samples of patients with idiopathic pulmonary fibrosis^46^. Based on the above data, we hypothesize that the up-regulation of miRNAs upon dysbindin deletion could be a negative feed-back process, aimed at attenuating TGF-β signaling linked to lung fibrosis^46^. In this perspective, miRNAs found up-regulated in the Dys^−/−^ retina deserve to be analyzed in pulmonary exudates of HPS-7 patients and/or animal models of pulmonary fibrosis. Moreover, these miRNAs could be further developed as pharmacological targets for treatment of pulmonary fibrosis in HPS-7. Additionally, we analyzed retinal staining of upstream effectors of TGFβ signaling pathway (TGFβ1, TGFβRI and TGFβRII), and we found a significant increase of TGFβRII staining in retinal ganglion cell layer of Dys^+/−^ and Dys^−/−^ mice, compared to control wild type mice. In physiological conditions retinal TGFβRII is localized in the retinal ganglion cell layer. TGFβRII depletion was found to be detrimental on retina development and function, leading to a low b-wave amplitude and to a degeneration of proximal retinal neurons^47^ (amacrine and ganglion cells^48^). On the basis of previous reports, TGFβRII expression levels can modulate the intensity of TGFβ1 signaling (smad or smad-independent signaling)^49^, in accordance with comorbidity linked to fibrosis in Hermansky Pudlak 7 patients^7–9,35^.

Furthermore, bioinformatic analysis, with the Tarbase algorithm, evidenced that miR-101-3p and miR-186-5p could target genes involved in LTP and LTD, the basic processes of neuronal plasticity that are affected in neurodevelopmental disorders^50^, consistently with the link between dysbindin dysfunctions and inefficient neuronal plasticity^2,51^. Dysbindin was found to regulate the expression of NMDA receptors, regulating hippocampal LTP in mice^31^. The *DISC1* “disrupted in the schizophrenia 1 gene” when mutated increases the risk of schizophrenia in humans^52,53^, and *DISC1* mutations impair LTP and LTD in mice^54^. DISC1 interacts with dysbindin influencing its own stability; moreover, DISC1 regulates neurite outgrowth induced by dysbindin^55^. Interestingly, some reports support the evidence that LTP occurs also at excitatory retinal synapses, formed by bipolar cells and retinal ganglion cells, in the developing retina^56^. Besides regulation of synaptic plasticity, dysbindin has been associated with various mechanisms underlying synaptic function^57–59^ and the regulation of dopamine and glutamate signaling in the brain^5,38,60^. Moreover, *in vitro* experiments suggest that dysbindin suppresses DA release^61^ and cultured neurons from Dys^−/−^ mice show increased cell surface expression of the dopamine D2 receptor, due to an increase of receptor membrane insertion^16^.

It is noteworthy that some dysbindin gene variants have been linked to visual dysfunctions^6,14^, which in turn were linked to a-wave and b-wave ERG aberrations^38,39^. Dopaminergic signaling and GABAergic transmission regulate adaptive mechanisms in the retina, in response to light brightness modifications. These two systems controls the light adaptation process in the retina, relying on rod bipolar cells sensitization and desensitization in dim-light or bright light conditions, respectively^36,37^. Moreover, glutamatergic and dopaminergic systems were found to be linked in retinal light adaptation^62,63^. Therefore, similar to effects in the brain, dysbindin mutations could affect the retina by altering the dopaminergic signaling and retinal synaptic plasticity. This hypothesis was supported by bioinformatic analysis, which predicted that dysregulated miRNAs in the retina of Dys^−/−^ mice could modulate dopaminergic, GABAergic and glutamatergic signaling, by targeting genes belonging to “amphetamine addiction”, “morphine addiction” and “glutamatergic synapse” pathways. Furthermore, it has been demonstrated that decreased retinal DA levels contributed to early visual and retinal dysfunction^64^. Furthermore, *in vitro* studies show that antipsychotics increase light sensitivity of retinal ganglion cells (RGCs) and this effect can be explained by D2 receptor antagonism^65^. Previous data clearly showed that the block of dopamine D1 receptors increases the b-wave amplitude^66^. This indicates that endogenous DA decreases the amplitudes of the ERG waves by acting through D1 receptors^66^. Moreover, it was found that the dopaminergic system could bi-directionally modulate the ERG response; i.e. the excitatory action on the b-wave could be mediated mainly by D2 receptors, while the suppressive effect upon the d-wave is mediated mostly by D1 receptors, which is counteracted by the dopamine excitatory action mediated through D2 receptors^67^. Furthermore, oscillatory potentials are regulated mainly by dopaminergic neurons, but also by GABAergic and glutamatergic system^68^. We analyzed the retinal function of Dys^−/−^ mice and found a significant increase in the b-wave, OP2 and OP3, compared to WT mice. This result is consistent with the hypothesis that dysbindin deletion could affect the dopaminergic signaling in the retina of Dys^−/−^ mice, due to an imbalance of DA retinal neurotransmission, similarly to what was found in neurons, where dysbindin siRNA treatment increased D2 receptor signaling along with decreased D2 receptor internalization^69^.

In conclusion, our findings on retinal miRNAs dysregulation in Dys^−/−^ mice, along with retinal dysfunction data, suggest that the Dys^−/−^ mouse represents a good paradigm for HSP-7, beyond a schizophrenia model as previously demonstrated^11,17,19^. Our data suggest that retinal function in ocular albinism could be modulated by miRNAs and also by dopaminergic tone. Further studies need to be carried out to directly assess dopaminergic signaling in the retina of  Dys^−/−^ mice (e.g. ERG measurements on Dys^−/−^ mice treated with dopaminergic agonists or antagonists). Finally, our findings support the hypothesis that pharmacological modulation of dysregulated miRNAs could be potentially relevant in clinical practice to counteract visual acuity reduction, and possibly pulmonary fibrosis in HSP-7 patients.

## Methods

### Bioinformatic miRNA prediction

High-throughput expression analysis of miRNAs is expensive and may requires a large number of samples, and post-hoc validation through quantitative real-time PCR^28^; for this reason, we first carried out an integrated bioinformatic approach to select miRNAs potentially dysregulated in Dys^−/−^mice. The first step included the prediction of newly disrupted or created miRNA-binding sites upon mutation of the dysbindin gene, through access to the miRSNPs database^70^. Predictions were carried out by filtering for European Caucasian ancestry. In order to decrease the false discovery rate of predictions, we mined at microRNA.org^71^ miRNAs that can regulate genes associated with albinism, such as: *TYR, TYRP1, OCA2, SLC45A2, DTNBP1, PAX3, MITF, SRY, SOX10, EDNRB*, and *EDN3*. Another bioinformatic analysis was carried out with the Diana miRPath v 3.0 tool^29^ to predict a focused miRNA set, to be further analyzed in WT, Dys^+/−^ and Dys^−/−^ mice. Targets were predicted applying the Tarbase algorithm. Pathways putatively dysregulated by miRNAs, listed in Tables 1 and 2, were rescored on the basis of their regulatory capability on the following signaling pathways and according to literature search data (pathway AND albinism; pathway AND neurodevelopment): “TGF-β1”^72^, “Protein Processing”^1,2,32^, “Fatty acid elongation”^73,74^, “endocytosis”^1,2^, “ubiquitin”^75^, “TNF signaling”^76^, “RNA transport”^1,2,77^, and “actin cytoskeleton”^78,79^. A post-hoc bioinformatic analysis was carried out with the Diana miRPath v 3.0 tool on the six out of 13 miRNAs, that were found to be dysregulated in retinal samples of Dys ^+/−^ and Dys^−/−^, compared to WT mice. In this case we iteratively used the algorithms Tarbase, microT-CDS and Targetscan^30^. If the Tarbase algorithm is applied, Diana miRPath predictions recall experimentally validated microRNA:RNA interactions, while microT-CDS and Targetscan rely on prediction and scoring of miRNA:RNA interactions^30^. Diana miRpath provided enriched depiction of KEGG pathways targeted by dysregulated miRNAs (KEGG permission n° 190309)^80^.

### Animals

All experimental procedures were approved by the IACUC of the University of Catania (approval # 640/2017-PR). Procedures were carried out in accordance to the Association for Research in Vision and Ophthalmology (ARVO) Statement for the Use of Animals in Ophthalmic and Vision Research. Dys^−/−^, Dys^+/−^ and WT mice used in this study, were produced using a heterozygous (Dys^+/−^) breeding strategy as previously described^11,19^. Wild-type C57BL/6 J mice were purchased from Envigo (San Pietro al Natisone, Udine, Italy). Six-month-old male Dys mutant mice (Dys^+/−^ and Dys^−/−^) and their wild-type littermates (Dys^+/+^) were used (N = 18 per genotype). N = 6 mice per genotype were used for miRNA expression analysis, while N = 12 per genotype animals were used for ERG and immunohistochemical assessment. All mice were genotyped using a duplex polymerase chain reaction (PCR) as previously described^10,14^. Primers for the WT gene, yielding a PCR product of 472 base pairs, were 5′-AGCTCCACCTGCTGAACATT-30 and 50-TGAGCCATTAGGAGATAAGAGCA-3′. Primers for the Dys mutant gene, yielding a product of 274 base pairs, were 5′-TCCTTGCTTCGTTCTCTGCT-3′ and 5′-CTTGCCAGCCTTCGTATTGT-3′. The 472-base-pair product was detected only in Dys^+/+^ and Dys^+/−^ mice, while the 274-base-pair product was detected only in the Dys^+/−^ and Dys^−/−^ mice. One-week prior ERG analysis, mice were moved from the animal colony room to a climate-controlled holding room (21 ± 1 °C), weighed, singly housed, and maintained on a 12-hour reverse light/dark cycle with free access to food and water. After sacrifice by means of cervical dislocation, eye globes were excised, and retina collected. Plasma samples were obtained, through intracardiac puncture, immediately after sacrifice of the mice.

### Extraction and qPCR of miRNAs

Total RNA was isolated from retina samples with TRIzol reagent (Thermo Fisher Scientific, Boston, MA, USA), according to the manufacturer’s instructions. Serum samples were centrifuged at 300 × *g* for 15 minutes at 4 °C to remove circulating cells and/or debris. Total RNA was isolated from 200 μl serum by using the Qiagen miRNeasy mini kit (Qiagen, Hilden, Germany), according to the Qiagen supplementary protocol for purification of small RNAs from serum and plasma; RNA was finally eluted in 45 μl of RNAse-free water and quantified by GenQuant pro spectrophotometer (Biochrom, Cambridge, UK). MiRNA expression analysis was performed through TaqMan assays. Reverse transcription of miRNAs was performed by TaqMan MicroRNA Reverse Transcription Kit (Thermo Fisher Scientific, Boston, MA, USA); the resulting miRNA-specific cDNA was amplified with the TaqMan microRNA Assays (Thermo Fisher Scientific, Boston, MA, USA) and the TaqMan Universal Master Mix II, no UNG (Thermo Fisher Scientific, Boston, MA, USA), according to the manufacturer’s instructions. MiR-16 was used as endogenous control for both retina and serum^81,82^. Real Time PCR reactions were performed on a 7900 HT Fast Real Time PCR System (Applied Biosystems, Monza, Italy). Differentially expressed miRNAs were identified by applying an unpaired T test (p-value ≤ 0.05); expression fold changes were calculated by the 2^−ΔΔCt^ method. PCR experiments followed MIQE guidelines.

### Tissue preparation for immunohistochemical staining

Eyes were enucleated and processed as previously described by Castorina A. *et al*.^83^. For each eye different sections where used for TGFβ1 or TGFβ Receptor I or TGFβ Receptor II staining. Immunohistochemical analysis was performed in accordance with the standard avidin–biotin complex (ABC) method. Briefly, sections were incubated with TGFβ1 (Abcam, #ab92486; 1:100) or TGFβ Receptor I (Abcam, #ab31013, 1:100) or TGFβ Receptor II (Cell Signaling, #3713; 1:100). Therefore, sections were incubated with a 1:200 diluted biotinylated goat anti–rabbit IgG for 1 h at room temperature. The sections were then rinsed and treated with reagents from an ABC Kit and counterstained with hematoxylin. All sections were examined, and images were taken with a light microscope (Axiovert, Carl Zeiss Inc). Densitometric analysis was carried with ImageJ^84^. TGFβ Receptor II staining in the retinal ganglion cell layer was quantified as follows: i. images were converted in black and white; ii. blue channel colour was switched off; iii. grey scale measurements were then normalized for the corresponding area of retinal ganglion cell layer.

### Scotopic ERG analysis

Before ERG testing, mice were dark adapted overnight. Scotopic ERG was carried out accordingly to previous reports about scotopic b-wave amplitude increase in albinos^13^. In anesthetized mice, pupils were dilated with 0.5% atropine, the cornea was intermittently irrigated with saline solution to prevent clouding of the ocular media, and a heating pad was used to keep the body temperature at 38 °C. The ERG responses were recorded through silver/silver chloride corneal electrodes and a forehead reference electrode. A ground electrode was placed on the tail. Scotopic ERG responses, which primarily measures rod function, were evoked by a 1 log cd-s/m^2^ flash generated through a Ganzfeld stimulator (Biomedica Mangoni, Pisa, Italy). The electrodes were connected to a two-channel amplifier. Signals were amplified at 1,000 gain and bandpass filtered between 0.2 and 500 Hz before being digitized at 5 kHz rate with a data acquisition device (Biomedica Mangoni, Pisa, Italy). The amplitude of the a-wave was measured at a fixed time of 8 ms after stimulus onset to minimize contamination from contributions of non-photoreceptor cells^85^. The b-wave amplitude was measured from the trough of the a-wave to the peak of the b-wave. Mean amplitudes of a- and b-wave ERG responses were plotted. For each experimental condition, ERG analysis was performed on 12 eyes per group (2 eyes for each animal), 6 mice per experimental group. Data was analyzed with respect to several parameters: a-, b-wave amplitudes and amplitudes of oscillatory potentials. Peak a-wave amplitude was measured from baseline to the initial negative going voltage, whereas peak b-wave amplitude was measured from the trough of the a-wave to the peak of the positive b-wave. In order to determine the amplitude of the oscillatory potentials (OP1-OP5), the ERG was low pass filtered at 16.5 Hz (red trace in Fig. 3) and subtracted from the original ERG wave. To evaluate the amplitude of OPs, ERG responses recorded at light intensity of 1 log cd-s/m^2^ were digitally filtered with a bandpass of 65–300 Hz to eliminate the a- and b-wave interference and to avoid the 60 Hz line noise.

### Statistical analysis

Experimental groups were masked to investigators during sample analysis for miRNA expression, immunohistochemistry, and ERG assessment. Labels were unveiled after analysis by investigators that carried out genotype and tissue collection. For the ERG and immunohistochemistry analysis *in vivo* experiments, all values are expressed as mean ± SD (N = 12 eyes per group, 2 eyes per animal, 6 mice per group). The quantitative PCR for miRNAs expression analysis was carried out on a total of 12 retinas for each experimental group, two retinas from each animal were pooled (N = 6 samples; each run in triplicate). Circulating miRNAs were evaluated in 18 serum samples from 6 mice per each genotype (N = 6 per group; each run in triplicate). Three independent experimental animal sets were used in this study for miRNA expression analysis, immunohistochemistry and ERG assessment, respectively. Statistical significance was assessed by one-way ANOVA with Tukey-Kramer post-hoc test for multiple comparisons. Differences were considered statistically significant for p-values < 0.05. GraphPad Prism v.7 (GraphPad Software, La Jolla, CA, USA) was used for statistical analysis and graph-figure design.

## Supplementary information

Supplementary Information.

## Data Availability

The datasets analyzed during the current study are available from the corresponding author on reasonable request.
