# Supplementary material for: Retinal biomarkers and pharmacological targets for Hermansky-Pudlak syndrome 7
Source: Sci Rep. 2020 Mar 4;10:3972. doi: 10.1038/s41598-020-60931-5 (PMC7055265; doi:10.1038/s41598-020-60931-5)

# Retinal biomarkers and pharmacological targets for Hermansky-Pudlak syndrome 7

Giovanni Luca Romano[1,2†], Chiara Bianca Maria Platania[2†], Gian Marco Leggio[2,3†], Sebastiano Alfio Torrisi[2], Salvatore Giunta[2], Salvatore Salomone[2,3], Michele Purrello[2], Marco Ragusa[2,4], Cristina Barbagallo[2], Frank J Giblin[5], Rosa Mastrogiacomo[6], Francesca Managò[6], Maurizio Cammalleri[7], Francesco Papaleo[6], Filippo Drago[2,3], and Claudio Bucolo[2,3,5]*

**Table 1S. Pathways dysregulated in the Dys$^{-/-}$ retina – Tarbase prediction (miR-101-3p, miR-186-5p, miR-382-5p)**

| KEGG pathway | p-value | #genes | #miRNAs |
|---|---|---|---|
| **Protein processing in endoplasmic reticulum** | 5.23E-07 | 45 | 3 |
| **Endocytosis** | 5.23E-07 | 55 | 3 |
| Adherens junction | 5.23E-07 | 24 | 3 |
| Lysine degradation | 1.12E-06 | 14 | 3 |
| **TGF-beta signaling pathway** | 6.39E-06 | 22 | 2 |
| Proteoglycans in cancer | 4.00E-05 | 43 | 3 |
| Adrenergic signaling in cardiomyocytes | 0.000451986 | 30 | 3 |
| Colorectal cancer | 0.001121462 | 18 | 3 |
| Thyroid hormone signaling pathway | 0.001124907 | 27 | 3 |
| Renal cell carcinoma | 0.001124907 | 18 | 3 |
| Hippo signaling pathway | 0.001608411 | 35 | 3 |
| N-Glycan biosynthesis | 0.002511303 | 8 | 3 |
| **Long-term potentiation** | 0.003314069 | 20 | 2 |
| Citrate cycle (TCA cycle) | 0.005242178 | 10 | 3 |
| Rap1 signaling pathway | 0.005253159 | 41 | 3 |
| Bacterial invasion of epithelial cells | 0.005821178 | 20 | 3 |
| Axon guidance | 0.008547487 | 27 | 3 |
| Gap junction | 0.008547487 | 17 | 3 |
| **Long-term depression** | 0.008962452 | 14 | 2 |
| Pathways in cancer | 0.008962452 | 74 | 3 |
| Regulation of actin cytoskeleton | 0.009031618 | 40 | 3 |
| Ubiquitin mediated proteolysis | 0.010973081 | 33 | 3 |
| Sphingolipid signaling pathway | 0.013210665 | 30 | 3 |
| cAMP signaling pathway | 0.014889036 | 40 | 3 |
| mRNA surveillance pathway | 0.016670219 | 25 | 3 |
| GnRH signaling pathway | 0.016670219 | 21 | 3 |
| mTOR signaling pathway | 0.016825723 | 16 | 2 |

| | | | |
|---|---|---|---|
| Dorso-ventral axis formation | 0.023078633 | 9 | 3 |
| MAPK signaling pathway | 0.023078633 | 48 | 3 |
| Endometrial cancer | 0.0298516 | 14 | 3 |
| Insulin signaling pathway | 0.029975051 | 30 | 2 |
| Neurotrophin signaling pathway | 0.029975051 | 26 | 3 |
| **Melanogenesis** | 0.035735426 | 21 | 2 |
| Focal adhesion | 0.035735426 | 40 | 3 |
| Steroid biosynthesis | 0.036787678 | 4 | 2 |
| Central carbon metabolism in cancer | 0.037481481 | 13 | 3 |

**Figure 1S. MiR-101-3p and miR-186-5p can control melanogenesis.** Yellow genes are targets of miR-101-3p and miR-186-5p. This picture is the output of Diana miRPath software and recalls the KEGG pathway deposited in the database (KEGG permission n° 190309): https://www.genome.jp/kegg/pathway.html

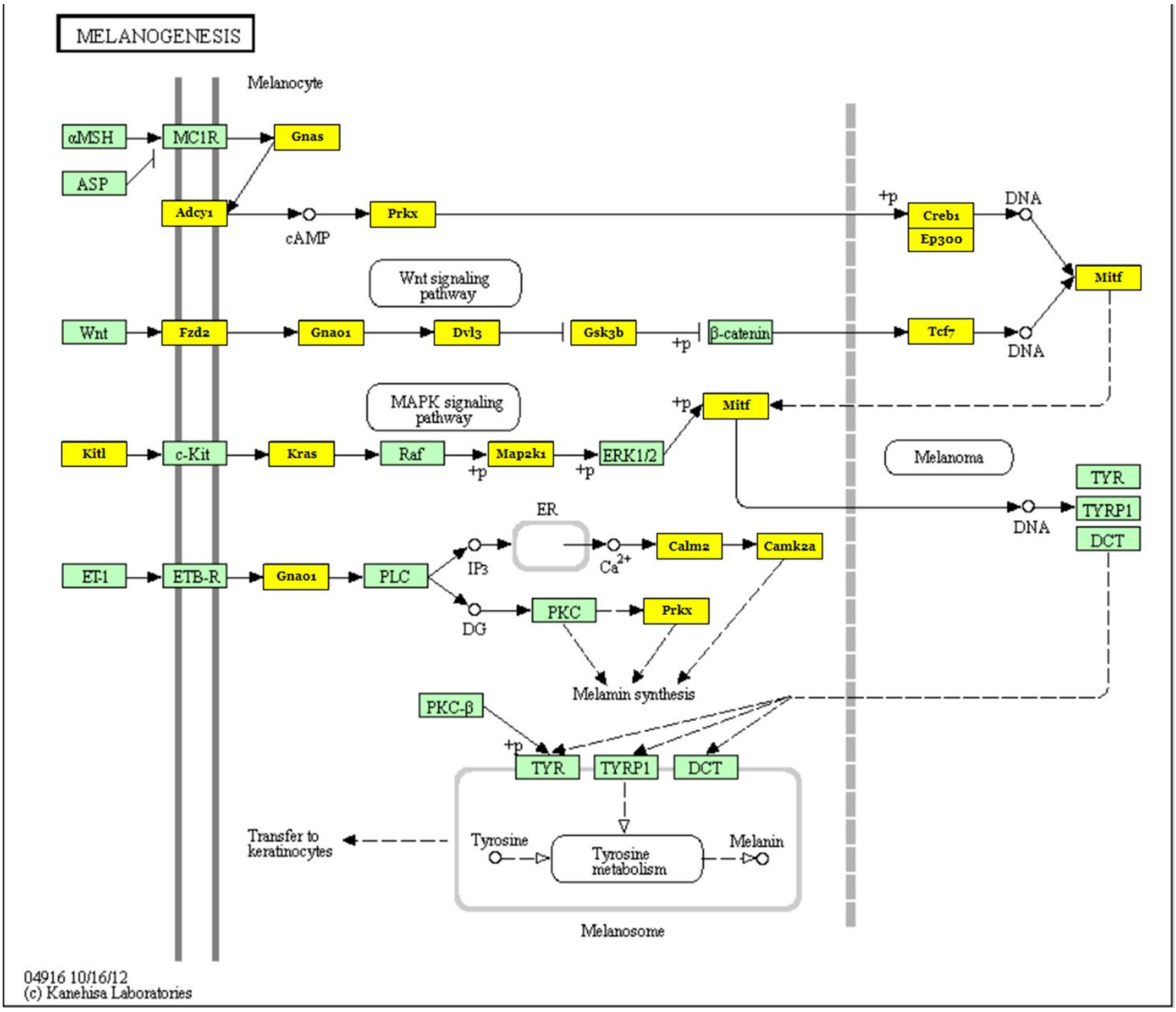

Supplement: Supplementary file 1 — Supplementary Information. [file 41598_2020_60931_MOESM1_ESM.pdf]
